# Who Is My Neighbor?

**Published:** 1864-01

**Authors:** 


### WHO IS MY NEIGHBOR?

Thy neighbor? It is he whom thou
Hast power to aid and bless—
Whose aching heart, or burning brow
Thy soothing hand may press.

Thy neighbor? 'tis the fainting poor;
Whose eye with want is dim,
Whom hunger sends from door to door—
Go thou, and succor him.

Thy neighbor? 'tis that weary man
Whose years are at their brim,
Bent low with sickness, cares, and pain—
Go thou and comfort him.

Thy neighbor? 'tis the heart bereft
Of every earthly gem—
Widow and orphan, helpless left—
Go thou, and shelter them.

Thy neighbor? yonder toiling slave,
Fettered in thought and limb,
Whose hopes are all beyond the grave—
Go thou, and ransom him.

Whene'er thou meet'st a human form
Less favored than thine own,
Remember 'tis thy neighbor-worm,
Thy brother or thy son.

Oh! pass not, pass not heedless by—
Perhaps thou canst redeem
This breaking heart from misery—
Go, share thy lot with him."

